# Energy Landscapes and Catalysis in Nitric-oxide Synthase*

**DOI:** 10.1074/jbc.M114.548834

**Published:** 2014-03-07

**Authors:** Anna Sobolewska-Stawiarz, Nicole G. H. Leferink, Karl Fisher, Derren J. Heyes, Sam Hay, Stephen E. J. Rigby, Nigel S. Scrutton

**Affiliations:** From the Manchester Institute of Biotechnology and Faculty of Life Sciences, University of Manchester, Manchester M1 7DN, United Kingdom

**Keywords:** Calmodulin, Enzyme Kinetics, Flavoproteins, Nitric-oxide Synthase, Protein Dynamics, PELDOR Spectroscopy, Free Energy Landscapes, High Pressure Stopped Flow

## Abstract

Nitric oxide (NO) plays diverse roles in mammalian physiology. It is involved in blood pressure regulation, neurotransmission, and immune response, and is generated through complex electron transfer reactions catalyzed by NO synthases (NOS). In neuronal NOS (nNOS), protein domain dynamics and calmodulin binding are implicated in regulating electron flow from NADPH, through the FAD and FMN cofactors, to the heme oxygenase domain, the site of NO generation. Simple models based on crystal structures of nNOS reductase have invoked a role for large scale motions of the FMN-binding domain in shuttling electrons from the FAD-binding domain to the heme oxygenase domain. However, molecular level insight of the dynamic structural transitions in NOS enzymes during enzyme catalysis is lacking. We use pulsed electron-electron double resonance spectroscopy to derive inter-domain distance relationships in multiple conformational states of nNOS. These distance relationships are correlated with enzymatic activity through variable pressure kinetic studies of electron transfer and turnover. The binding of NADPH and calmodulin are shown to influence interdomain distance relationships as well as reaction chemistry. An important effect of calmodulin binding is to suppress adventitious electron transfer from nNOS to molecular oxygen and thereby preventing accumulation of reactive oxygen species. A complex landscape of conformations is required for nNOS catalysis beyond the simple models derived from static crystal structures of nNOS reductase. Detailed understanding of this landscape advances our understanding of nNOS catalysis/electron transfer, and could provide new opportunities for the discovery of small molecule inhibitors that bind at dynamic protein interfaces of this multidimensional energy landscape.

## Teaser

**Background:** Protein domain dynamics and calmodulin binding are implicated in regulating electron flow in NO synthase.

**Results:** A dynamic conformational landscape important for enzyme catalysis is demonstrated.

**Conclusion:** NO synthesis requires a complex landscape of conformations, with calmodulin as a key driver of chemistry through modulation of the dynamic landscape.

**Significance:** Detailed understanding of conformational landscapes provides new opportunities for inhibitor discovery targeted at the dynamic interfaces.

## Introduction

**FIGURE 1.**
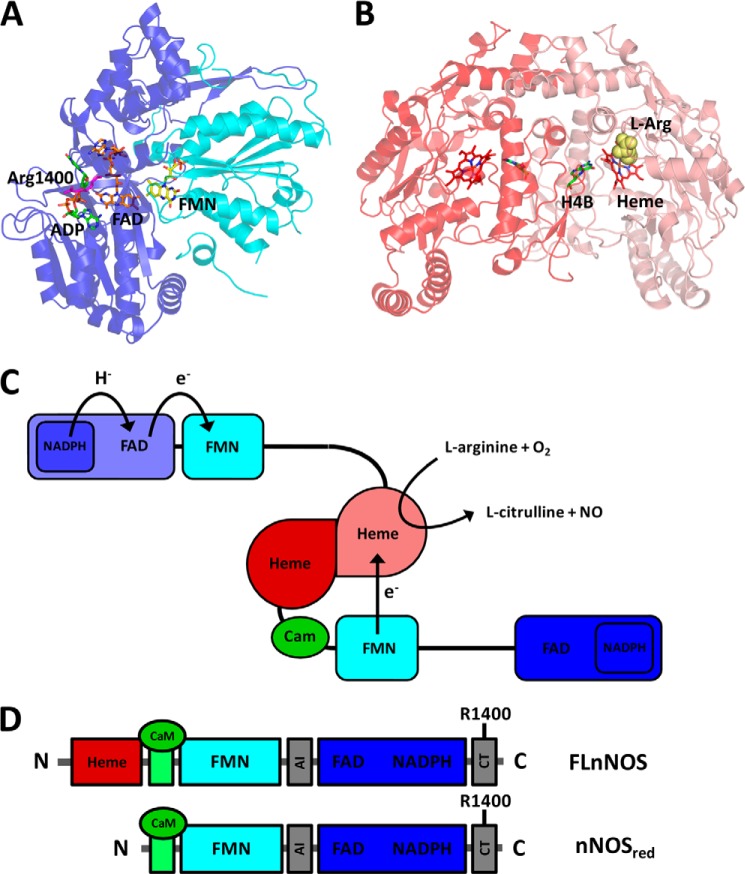
**Structural organization of nNOS.** *A*, crystal structure of the nNOS diflavin reductase domain with bound ADP (PDB 1TLL) (10). The FAD-binding subdomain is highlighted in *blue*, and the FMN-binding subdomain is highlighted in *cyan*. The distance between the FAD and FMN cofactors is 15.5 Å. *B*, crystal structure of the nNOS oxygenase dimer with bound l-arginine (PDB 1ZVL) (52). The oxygenase and reductase domains are connected via a CaM-binding region. *C,* schematic representation of a NOS homodimer in the non-productive state without CaM bound, and its productive state with CaM bound. *D*, recombinant nNOS constructs used in this study. The CaM-binding domain is highlighted in *green*, and the two regions that harbor the autoinhibitory insert (*AI*) and the C-terminal tail (*CT*) regulatory sequences are highlighted in *gray*. FLnNOS is the full-length nNOS protein, including the N-terminal oxygenase domain, CaM-binding region, and the C-terminal diflavin reductase domain. nNOS_red_ comprises the CaM-binding region and the C-terminal diflavin reductase domain (residues 695–1429). *H4B*, tetrahydrobiopterin.

Nitric oxide (NO) is a small, readily diffusible molecule with a central role in several key physiological processes, including blood pressure regulation, neurotransmission, and immune response (1). NO is produced by different tissue-specific NO synthases (NOS). NOS enzymes are monooxygenases that catalyze the NADPH- and oxygen-dependent conversion of l-arginine to citrulline and NO, through a series of complex electron transfer reactions that are influenced by calmodulin (CaM)^3^ binding and protein dynamics (2–5). There are three mammalian NOS isoforms: neuronal (nNOS), endothelial (eNOS), and inducible (iNOS). CaM reversibly activates nNOS and eNOS in a Ca^2+^-dependent manner; iNOS is constitutively activated by CaM binding regardless of intracellular Ca^2+^ concentrations (2). All three isoforms of NOS are complex homodimeric enzymes. Each monomer comprises an N-terminal oxygenase domain containing heme and tetrahydrobiopterin cofactors, an l-arginine binding site, and a C-terminal diflavin reductase domain that contains binding sites for NADPH and the cofactors FAD and FMN; both domains are separated by a CaM binding region (Fig. 1). NOS enzymes receive electrons at the FAD cofactor by hydride transfer from NADPH. These electrons are then shuttled to the heme oxygenase domain via the FMN cofactor. Electron transfer is regulated by several unique protein regulatory inserts in the C-terminal reductase domain, including an autoinhibitory peptide insert (6) and a C-terminal peptide tail (7). In the absence of CaM, NADP(H) binding is proposed to lock the nNOS reductase domain in a conformation that restricts motion of the FMN-binding domain. In this locked conformation, electron transfer from FMN to the oxygenase domain is inhibited, as is formation of NO (8). This “conformational lock” is thought to be mediated by Arg-1400 located in the C-terminal tail (9), the effects of which are relieved by CaM binding (8).

Despite a wealth of available spectroscopic, kinetic, and structural data there remains little information at a molecular level on conformational transitions coupled to electron transfer and catalysis. A binary model involving “open” and “closed” forms of NOS reductase has been proposed from the “static” crystal structure of nNOS reductase (10). Kinetic and solution spectroscopy studies have supported this model (3, 4, 11). This simple model has been extended in the context of full-length NOS to suggest shuttling of the FMN domain between the NADPH/FAD and oxygenase domains. This extended model therefore implies conformational control of interdomain (FAD to FMN, and FMN to heme) electron transfer (2, 12, 13). CaM is known to activate NO synthase activity by a proposed movement of the FMN domain closer to the heme oxygenase domain to increase the probability of electron transfer (14). Domain interaction surfaces have been mapped by hydrogen-deuterium exchange mass spectrometry for iNOS, highlighting the interfaces between the heme oxygenase domain, the FMN domain, and CaM. This has provided important new insights into conformational properties that impact on iNOS activity (15).

Conformational sampling and its importance to biological electron transfer has been emphasized in recent years (16–18). Flavoprotein crystallography has contributed to this emerging picture of the importance of conformational sampling in dynamic redox systems (10, 19–21). Pulsed electron-electron double resonance (PELDOR) spectroscopy enables distance measurements between paramagnets; flavin semiquinones are natural biological paramagnets that are ideally suited to PELDOR investigations (22). This approach has been used to investigate the natural conformational landscapes of dynamic flavoproteins, including cytochrome P450 reductase (CPR) and methionine synthase reductase (23, 24). In addition, changes in hydrostatic pressure can perturb the free energy landscapes of dynamic multidomain enzymes (23, 24) and can be used to map the “fitness” (*i.e.* rate of electron transfer/enzyme turnover) in relationship to the conformational/free energy landscape (23, 25, 26). A combination of PELDOR spectroscopy and “pressure fitness” measurements should provide unique spatial-temporal information that will enhance the molecular level understanding of biological redox catalysis. Here we take such an approach to provide much needed insight into the relationship between domain dynamics and electron transfer/catalysis in the multidomain enzyme nNOS.

## MATERIALS AND METHODS

### 

#### 

##### Recombinant Protein Expression and Purification

The native rat neuronal NOS reductase domain (nNOS_red_) and the R1400E variant were expressed from plasmid pCRNNR (27) in *Escherichia coli* BL21(DE3) and purified by 2′,5′-ADP-Sepharose affinity chromatography followed by Q-Sepharose anion-exchange chromatography as described previously (27, 28). Protein concentrations were determined at 454 nm using an extinction coefficient of ϵ_454_ = 21.6 mm^−1^ cm^−1^ (29). Native rat full-length His_6_-tagged nNOS and full-length His_6_-tagged R1400E were expressed in *E. coli* BL21(DE3) from a modified pCWori plasmid (9) and purified by nickel-nitrilotriacetic acid-agarose affinity chromatography and 2′,5-ADP-Sepharose affinity chromatography according to published methods (30). Protein concentrations were determined at 444 nm in the presence of carbon monoxide (CO), using a molar extinction coefficient of 74 mm^−1^ cm^−1^ (*A*_444_–*A*_500_) for the ferrous heme-CO adduct (31). The gene encoding mammalian CaM was cloned between the NdeI and SacI restriction sites of the pCOLADuet-1 plasmid (Novagen). Recombinant CaM was expressed in *E. coli* and purified using a single phenyl-Sepharose hydrophobic interaction chromatography step as described before (32). Protein concentrations were determined at 276 nm using an extinction coefficient of ϵ_276_ = 3.006 mm^−1^ cm^−1^. The CaM T34C variant was constructed using the CaM containing pCOLADuet-1 plasmid as a template according to the QuikChange method (Stratagene). The CaM cysteine variant was expressed and purified as mentioned above.

##### Anaerobic Sample Preparation

Samples for anaerobic experiments were prepared and handled anaerobically in a Belle Technology glovebox under nitrogen atmosphere in which oxygen levels were kept below 2 ppm. All buffers and solutions were degassed by bubbling with oxygen-free nitrogen prior to entering the glove box, and left overnight to equilibrate, to ensure removal of all traces of oxygen. The nNOS was purified in a partially reduced form and had to be oxidized prior to each experiment by adding a few grains of the oxidant potassium ferricyanide in the anaerobic box. The protein solution was applied immediately onto a desalting column pre-equilibrated with the desired anaerobic buffer. Thus, this step resulted in the removal of ferricyanide as well as achieving the transfer of nNOS into the required anaerobic buffer system.

For redox, potentiometry external redox mediators (benzyl viologen, 2-hydroxy-1,4-naphthaquinone, methyl viologen, and phenazine methosulfate) were added to the protein solution to promote one-electron transfer equilibria. The protein solutions were titrated chemically using sodium dithionite as the reductant (33). Sodium dithionite was delivered in small powder aliquots. Spectra were recorded using a Cary UV-50 Bio UV-visible scanning spectrophotometer. For titration of the nNOS_red_, absorbance values at 456 nm (near the absorption maximum for the oxidized flavins in both nNOS_red_ CaM^−^ and nNOS_red_ CaM^+^) and 592 nm (near the absorption maximum for the blue semiquinone form of the flavins) were analyzed. Samples (200–250 μl) of enzyme were withdrawn for EPR spectroscopic analysis. The samples were placed in standard 4.0-mm quartz EPR tubes and sealed with a rubber Suba-Seal inside the glovebox, where they were immediately removed and frozen in liquid nitrogen. Samples were stored in liquid nitrogen to prevent reoxidation until they were analyzed. All EPR samples were prepared in 50 mm potassium phosphate buffer (pH 7.0) containing 30% (v/v) glycerol. The concentrations of nNOS_red_ and full-length nNOS in CW EPR samples, were typically 70 μm. The concentrations of nNOS_red_ and R1400E nNOS_red_ in PELDOR samples, both CaM-bound and CaM-free, were typically 350 μm. The concentrations of full-length nNOS in PELDOR samples, both CaM-bound and CaM-free, were 410 and 290 μm, respectively. The concentration of NADP^+^, if added, was ∼400 μm.

##### Electron Paramagnetic Resonance

**FIGURE 2.**
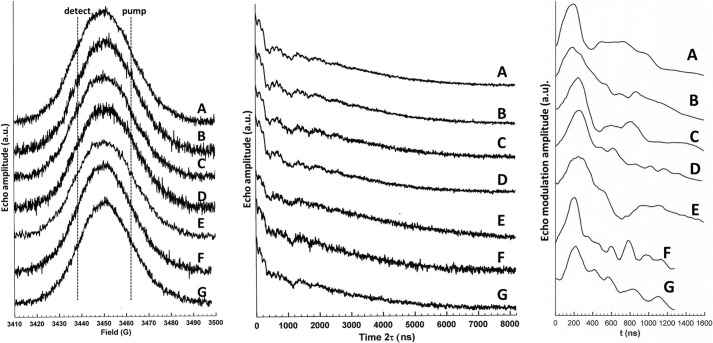
**X-band pulsed EPR measurements on the disemiquinoid forms of nNOS.** *Left*, X-band echo-detected pulse field swept spectra. The approximate field positions of the PELDOR pump and detection pulses are shown. Recorded at 80 K using a soft pulse spin echo sequence π/2-τ-π-τ-acquire with π/2 = 60 ns and τ = 800 ns. *Middle*, X-band 2-pulse spin echo decay measurements taken at the detection field (see *left panel*). Recorded at 80 K using a hard pulse spin echo sequence π/2-τ-π-τ-acquire with π/2 = 16 ns and τ = starting at 200 ns and incrementing in 8-ns steps. *Right*, refocused echo modulation patterns arising from the X-band PELDOR experiments. See “Materials and Methods” for the experimental parameters. Data were not baseline corrected. *A*, nNOS_red_; *B*, nNOS_red_ + NADP^+^; *C*, nNOS_red_ + CaM; *D*, nNOS_red_ + CaM + NADP^+^; *E*, R1400E nNOS_red_ + CaM + NADP^+^; *F*, full-length nNOS; *G*, full-length nNOS + CaM.

All EPR spectra were obtained using a Bruker ELEXSYS E500/E580 spectrometer operating at X-band. Temperature control for pulsed measurements was achieved using an ESR935 cryostat connected to an ITC503 temperature controller. Four pulse ELDOR spectra were recorded using a π/2-*T*-π-*T*+τ-π-τ-acquire sequence with *T* = 200 ns and τ = 960 ns. The π pulse length was 32 ns. The “fourth” pulse, a π pulse applied at the pump microwave frequency (75 MHz “upfield” of the detection frequency, Fig. 2, *left*), was incremented in 16 ns steps during the *T*+τ period starting at 100 ns after the second π pulse. The interval τ was limited to 960 ns due to the spin-spin relaxation, phase memory time *T_M_*, of the flavosemiquinones that precluded detection of the refocused echo at longer values of τ (Fig. 2, *middle*). If the timing of the fourth pulse is indicated as *t* = 0 at 100 ns after the second π pulse and *t* is incremented thereafter, the modulation of the four-pulse ELDOR echo is given by *V*_4p_(*t*) = cos(ω_ee_(*t* − *T*))^37^, where ω_ee_ is the electron-electron coupling. All pulse experiments were recorded at 80 K and at a repetition frequency of 200 Hz due to the long electron spin lattice relaxation time of the flavosemiquinone radical. Attempts to prolong τ by lowering the temperature and thus lengthen *T_M_* were prevented by the impact on the repetition rate (governed by the electron spin lattice relaxation time *T*_1_*e*), which led to prohibitively slow repetition rates. Inter-electron dipolar couplings were determined using Fourier transform of the baseline corrected four-pulse ELDOR data to produce dipolar spectra. The raw PELDOR data (Fig. 2, *right*) were baseline corrected using a third order polynomial, then a Hamming window function was applied to increase signal to noise following the subsequent Fourier transform and drive the decay function to completion, thus avoiding truncation effects. Each data set was then 0 filled to the 1024 point prior to Fourier transformation.

The dipole-dipole coupling between two electronic magnetic moments of the flavin semiquinone centers is related to the distance between them (Equation 1) (23, 34),

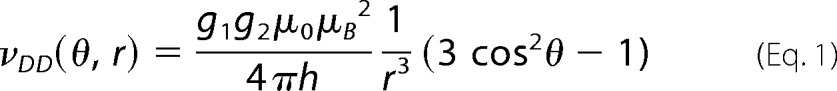
 where *g*_1_ and *g*_2_ are the *g* values of the two spins, *r* is the distance between them, and θ is the angle between the inter-spin vector and the applied magnetic field. Therefore, determining the dipole-dipole coupling between the two flavinsemiquinones in nNOS provides a means of quantifying differences in inter-flavin distances because the observed discrete dipolar coupling, υ_DD_, can be converted to discrete inter-flavin distances using Equation 1. Only the intense “perpendicular” (θ = 90°) components of the powder pattern lines produced by this experiment were used. The “parallel” components (θ = 0°) of the powder pattern are much weaker (∼10% of the intensity of the perpendicular components) and, therefore provide a less accurate estimation of υ_DD_, and hence the distance (34).

##### High Pressure Stopped-flow

High pressure stopped-flow experiments were performed using a Hi-Tech Scientific HPSF-56 high pressure stopped-flow spectrophotometer. All experiments were performed in 40 mm HEPES buffer (pH 7.6) supplemented with 150 mm NaCl and 10% (v/v) glycerol at 25 °C. Flavin reduction by NADPH was monitored at 458 nm, a heme isosbestic point. The reaction was started by mixing 5 μm nNOS with 100 μm NADPH (final concentrations) under anaerobic conditions in the presence or absence of 25 μm CaM and 1 mm Ca^2+^. Under these conditions the observable rates are independent of the coenzyme concentration (27). Single turnover NO formation was triggered by rapid mixing of oxidized or pre-reduced NOS in the presence of 2 mm l-arginine and 10 μm tetrahydrobiopterin, with 100 μm NADPH or CaM/Ca^2+^, respectively. NO formation was monitored at 401 nm (Δϵ_401_ = 38 mm^−1^ cm^−1^) (35) using 10 μm oxyhemoglobin. Steady-state activities were determined on assay mixtures containing catalytic amounts of nNOS, 2 mm l-arginine, 1 mm CaCl_2_, and 100 μm NADPH in the presence or absence of CaM in assay buffer. Steady-state NADPH oxidation rates were determined at 340 nm (ϵ_340_ = 6.22 mm^−1^ cm^−1^) and cytochrome *c* reduction at 550 nm (Δϵ_550_ = 21.1 mm^−1^ cm^−1^) in the presence of 10 μm cytochrome *c*. Superoxide formation was determined by performing the cytochrome *c* reduction assay in the presence or absence of superoxide dismutase (1–2 units ml^−1^). The pressure (*p*) dependence of the observed rate constants can be extracted from Equation 2 (23),

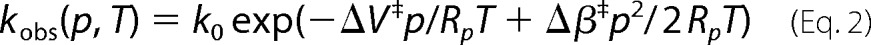
 where *R_p_* = 83.13 cm^3^ mol^−1^ bar K^−1^, *k*_0_ is the observed rate constant extrapolated to 0 bar, Δ*V*^‡^ is the apparent difference between the volume of the reactant and transition states, and Δβ^‡^ is the compressibility of the transition state: Δβ^‡^ = dΔ*V*^‡^/*dp*.

##### Fluorescence

The CaM T34C cysteine variant was labeled with the fluorescent dye Atto532-maleimide (Sigma) according to the manufacturer's instructions. Fluorescence measurements were performed in a FLS920 fluorometer (Edinburgh Instruments) fitted with an ISS high pressure cell. Samples contained 100 nm fluorescently labeled CaM, 100 nm NOS, and 300 μm CaCl_2_ or 500 μm EDTA in 40 mm HEPES buffer (pH 7.6) containing 10% glycerol, 150 mm NaCl, 2 mm l-arginine, 10 μm tetrahydrobiopterin, and 100 μm NADPH. The samples were excited at 536 nm and fluorescence emission spectra were recorded at 25 °C between 1 and 1750 bar, with 250 bar intervals.

##### Data Analysis

Analysis of spectral transients obtained from stopped-flow experiments and non-linear fitting of data were performed using Origin software (OriginLab). The PELDOR data were analyzed using the software package supplied with the instrument.

## RESULTS AND DISCUSSION

### 

#### 

##### Access to the Spatial Energy Landscape through Low Temperature PELDOR

**FIGURE 3.**
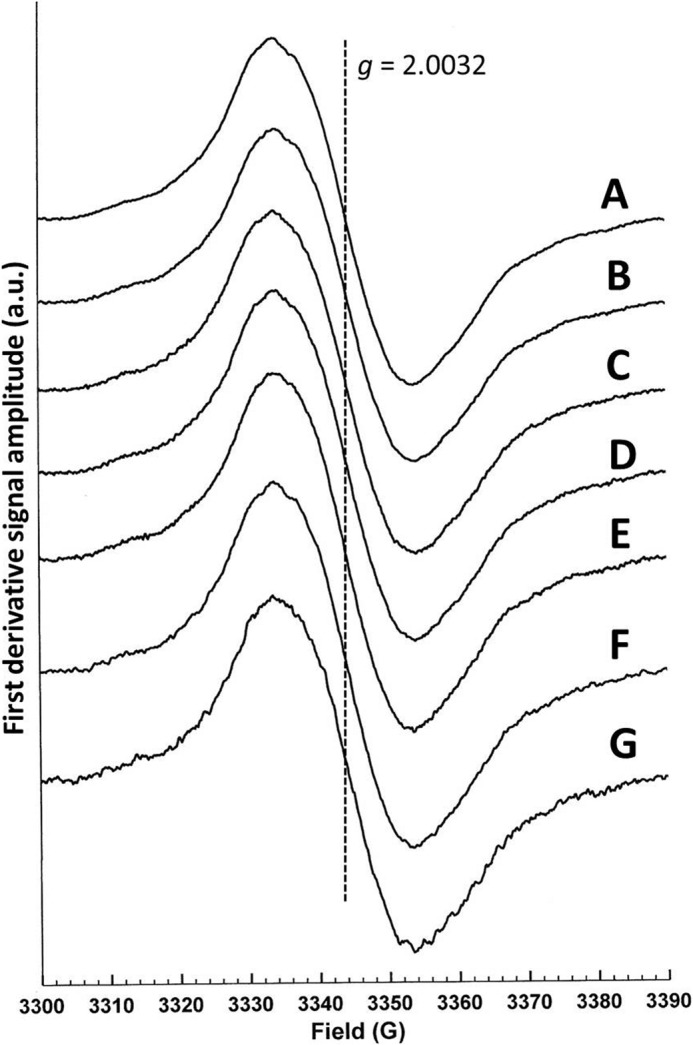
**The X-band continuous wave EPR spectra of the disemiquinoid forms of nNOS.** *A*, nNOS_red_; *B*, nNOS_red_ + NADP^+^; *C*, nNOS_red_ + CaM; *D*, nNOS_red_ + CaM + NADP^+^; *E*, R1400E nNOS_red_ + CaM + NADP^+^; *F*, full-length nNOS; and *G*, full-length nNOS + CaM. Experimental parameters: microwave power, 10 μW; modulation amplitude, 1 G; temperature, 80 K.

nNOS can be reductively titrated under anaerobic conditions to generate an equilibrium distribution of redox forms, of which only the flavin semiquinones can be observed using EPR spectroscopy (Fig. 3). One such form, the diflavosemiquinoid species, bears two unpaired electrons, one on each of the FMN and FAD cofactors. Here we used PELDOR spectroscopy of this diflavosemiquinone to measure distances between the FAD and FMN cofactors of the two-electron reduced nNOS diflavin reductase domain (nNOS_red_), full-length nNOS and nNOS_red_ R1400E. The effects of ligand (NADP^+^) and CaM binding on the energy landscape were also studied.

**FIGURE 4.**
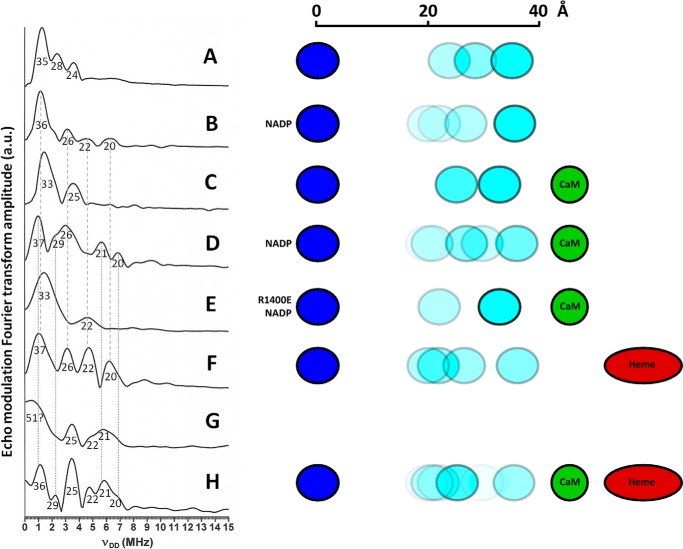
**Multiple nNOS conformations accessed by PELDOR spectroscopy.** Fourier transforms of the refocused echo modulation obtained from four-pulse ELDOR experiments on: nNOS_red_ (*A*), nNOS_red_ + NADP^+^ (*B*), nNOS_red_ + CaM (*C*), nNOS_red_ + CaM + NADP^+^ (*D*), R1400E nNOS_red_ + CaM + NADP^+^ (*E*), full-length nNOS (*F*), and full-length nNOS + CaM (*G*). *H*, the data of *G* resolution enhanced in post-acquisition processing. The *numbers under each line* in the data indicate inter-flavin distances in Å. The *vertical dashed lines* and *dotted lines* show the similarities between the data of *B* and *F*, and *C* and *H*, respectively. Experimental conditions are given under “Materials and Methods.” The schematics show the observed distance distribution between the FAD (*blue*) and FMN (*cyan*) semiquinones in each sample.

The PELDOR results represent the conformational distributions exhibited by the protein just before motion ceased because of lack of thermal energy at 80 K. These “trapped” conformations exist at the bottom of thermodynamic wells or minima. Fig. 4*A* shows the Fourier transform of the X-band four-pulse ELDOR data obtained from the diflavosemiquinone form of nNOS_red_ in the absence of calcium-calmodulin (CaM). Three maxima are evident indicating three dipolar coupling values and three corresponding inter-flavin distances of 35, 28, and 24 Å. This suggests quite a “rugged” conformational energy “landscape” at low temperature (80 K), with three energy minima each having a different inter-flavin distance. Upon addition of NADP^+^ to nNOS_red_ (Fig. 4*B*) the conformational energy landscape becomes yet more rugged with four ν_DD_ values and their conjugate distances were observed. However, the amplitudes of the spectrum contributions suggest that the protein conformation with the longest distance between FAD and FMN, 36 Å, is the most highly populated. The shortest inter-flavin distance evident in these data is 20 Å, shorter than observed without NADP^+^ but, as stated above, this conformation is not highly populated. CaM binds to nNOS and such binding promotes electron transfer and NO synthesis. The CaM-bound nNOS_red_ PELDOR data (Fig. 4*C*) represent the simplest and least rugged conformational energy landscape observed so far, exhibiting only two conformations with inter-flavin distances of 33 and 25 Å. This is in stark contrast to nNOS_red_ with bound CaM and NADP^+^ (Fig. 4*D*), which displays a very rugged conformational energy landscape of five detectable conformations and an equilibrium between these conformations that favors shorter inter-flavin distances. The distances obtained are summarized in Table 1.

**TABLE 1:** **Inter-flavin dipole-dipole coupling, ν_DD_, distances and relative integrals derived from the PELDOR data obtained from two electron reduced nNOS and nNOS_red_ shown in Fig. 4**

	ν_DD_	Distance, *r*	Relative integral
	*MHz*	Å	
WT nNOS_red_ CaM^−^	1.3, 2.4, 3.6	35, 28, 24	0.52, 0.28, 0.20
WT nNOS_red_ CaM^−^/NADP^+^	1.1, 3.2, 4.6, 6.2	36, 26, 22, 20	0.62, 0.20, 0.08, 0.10
WT nNOS_red_ CaM^+^	1.4, 3.6	33, 25	0.70, 0.30
WT nNOS_red_ CaM^+^/NADP^+^	1.0, 2.2, 3.0, 5.7, 6.8	37, 29, 26, 21, 20	0.36, 0.14, 0.21, 0.18, 0.11
R1400E nNOS_red_ CaM^+^	1.4, 3.6	33, 25	0.70, 0.30
R1400E nNOS_red_ CaM^+^/NADP^+^	1.4, 4.6	33, 22	0.87, 0.13
FL WT nNOS CaM^−^	1, 3.1, 4.7, 6.2	37, 26, 22, 20	0.46, 0.18, 0.20, 0.16
FL WT nNOS CaM^+^	0.5, 3.5, 4.7, 5.8 (res. enhanced, 1.1, 2.2, 3.4, 4.7, 5.8, 6.8)	51, 25, 22, 21 (res. enhanced, 36, 29, 25, 22, 21, 20)	0.69, 0.12, 0.05, 0.14

Care should be taken when the inter-flavin distances obtained by PELDOR measurements are compared with inter-flavin distances obtained from x-ray structures. The unpaired electron is delocalized over all three rings of the flavin semiquinone, thus giving the distance between a weighted mean of the unpaired electron density distribution, rather than the distance between two points. The center of the weighted mean of the density distribution is the C4a atom (36) and thus it is taken as the reference atom. The inter-flavin distance in the crystal structure of NADP^+^-bound nNOS_red_ (Fig. 1*A*, PDB 1TLL) (10) is 15.5 Å, in reasonable agreement with the observed shortest inter-flavin distances of 20 Å obtained from the PELDOR experiments for nNOS_red_. A similar agreement between PELDOR-derived distances for closed and open forms (19 *versus* 36 Å) and their respective crystal structures (15.4 *versus* 31.6 Å) was observed for CPR (23). Furthermore, in the absence of a structure for nNOS_red_ in its open form, the estimated longer inter-flavin distances (33–37 Å) from this study are in excellent agreement with those observed for CPR and methionine synthase reductase in related PELDOR studies (23, 24). A recent structure of an open form of CPR (with inter-flavin distances of 30–32 Å) in complex with its heme oxygenase redox partner demonstrates the catalytic relevance of this open conformation, and the observed FMN to heme distance of 6 Å implies direct electron transfer from FMN to heme (37).

Arg-1400 is located in the C-terminal regulatory sequence (Fig. 1*D*), where it interacts with the 2′-phosphate of NADPH, thereby regulating electron flow through nNOS. It is believed that the Arg-1400–2′-phosphate interaction is a means by which bound NADPH represses electron transfer into and out of the nNOS_red_ domain in the absence of CaM. This interaction enables the C-terminal tail to regulate a conformational equilibrium of the FMN module that controls its electron transfer reactions in both the CaM-free and CaM-bound forms of nNOS (9). In agreement with this, the PELDOR data obtained for nNOS_red_ R1400E in the presence of NADP^+^ and CaM (Fig. 4*E*) shows only two conformations, suggesting a conformational energy landscape almost identical to the native CaM-bound enzyme in the absence of NADP^+^. This supports the notion that the interaction of Arg-1400 with 2′-phosphate contributes to the change in conformational equilibrium.

The observation of inter-flavin distance distributions in full-length nNOS using PELDOR spectroscopy requires that we consider the role of the heme domain. The latter may not only influence the equilibrium between reductase domain conformations, but also the magnetic properties of the enzyme that impact on the outcome of the PELDOR experiment. The heme domain is cytochrome P450-like with a thiolate proximal ligand to the heme iron. However, unlike cytochromes P450, the iron is in the high spin state even at low temperature (38). Furthermore, reduction of the heme iron during the formation of the diflavosemiquinone leaves it in the paramagnetic (although EPR “invisible”) *S* = 2 high spin ferrous state, rather than creating a diamagnetic state. This high spin iron will decrease the *T_M_* (electron spin phase memory time) of the flavin semiquinones as they get closer to the heme, making their contributions to the PELDOR data weaker and more difficult to observe. Full-length nNOS shows a complex energy landscape with multiple conformational states as evident in the data of Fig. 4, *F* and *G*. In the absence of CaM, the inter-flavin distance distribution matches that exhibited by nNOS_red_ without CaM, but with NADP^+^ bound (compare with Fig. 4*B*). There is, however, a marked difference in the relative amplitudes of the states present in these two spectra (Fig. 4, *B* and *F*). In NADP^+^-bound nNOS_red_ without CaM, the distance distribution is dominated by the 36-Å inter-flavin distance, whereas in the full-length protein without CaM the distribution between conformations is more even. This may reflect the aforementioned influence of the heme iron on *T_M_*, leading to a reduction in the amplitude of the contribution from the conformation with a 36-Å inter-flavin distance, as in this conformation the FMN radical would be closer to the heme than in the other conformations observed. Nevertheless, these data suggest that the heme domain does influence the conformational equilibrium within the reductase domain. The presence of CaM gave rise to the data of Fig. 4*G*. This spectrum is unusually broad and weak, perhaps reflecting a change in the redox state of the heme as diflavosemiquinone formation requires a lower applied potential in the presence of CaM (11). Extensive data collection was used to compensate for the low intensity and a data processing protocol that incorporated a shifted Gaussian window function allowed for some resolution enhancement, the results of which are shown in Fig. 4*H* (note that Fig. 4, *F* and *G*, employ the same raw data and differ only in the application of the Gaussian window to *H*). Prior to resolution enhancement the data are dominated by a broad line corresponding to a distance of 51 Å, suggestive of an inter-flavin distance between cofactors on two different monomers as depicted in the “crystallographic dimer” (the x-ray crystal structure gives distances measured C4a to C4a of 49 Å for FMN to FMN, 39 Å for FAD to FMN, and 42 Å for FAD to FMN). It is important to note, however, that nNOS reductase is monomeric in solution. This broad line, however, appears to arise from an overlap of several contributions, which are separated in the resolution enhanced data. The latter, Fig. 4*H*, shows discrete lines equating to a distance distribution that suggests comparable conformational equilibria exist in full-length nNOS with CaM bound and nNOS_red_ with bound CaM and NADP^+^ (see Table 1). Prior to resolution enhancement, these discrete lines are overlapped by a broad component that appears to arise from ν_DD_ values of less than 0.5 MHz, part of which is still visible at the far left of Fig. 4*H*. CaM binding in the full-length protein shifts the equilibrium toward on average slightly longer inter-flavin distances (compare Fig. 4, *F* and *H*, where the 25 Å species is enriched in the latter). The landscapes, however, are qualitatively similar and there is significant population of more closed conformations of the reductase component (*i.e.* shorter FAD-FMN distances). This suggests conformational sampling of the FMN domain is required to enable electron transfer from the diflavin reductase domains to the heme oxygenase domain during catalysis. This may explain why electron transfer from FMN to heme, in the presence of CaM, is relatively slow (∼1 s^−1^), and limits overall catalysis. This notion is supported by a recent thermodynamic study of the heme reduction kinetics in nNOS (39), which suggested that the FMN-nNOS_oxy_ interaction in the presence of CaM is intermittent and/or transient.

Our PELDOR data provide direct access to the nNOS energy landscape for domain motions and identified conformations hitherto inaccessible by other structural and spectroscopic techniques. However, PELDOR only allows the study of the di-semiquinoid, or two-electron reduced form. Interestingly, fluorescence resonance energy transfer, small-angle x-ray scattering, and nuclear magnetic resonance studies of CPR (40–42) indicate that the equilibrium between the closed and open conformations depends on the redox state of the enzyme as well as the binding of coenzyme. Coenzyme binding shifts the conformational equilibrium of CPR to a more closed conformation, ideal for inter-flavin ET, whereas two-electron reduction of CPR after hydride transfer from NADPH leads to a more open conformation, ready for interaction with a redox partner. The question remains as to whether this redox-driven conformational control also occurs in nNOS, as our PELDOR data demonstrate that CaM provides an additional level of conformational control by shifting the conformational equilibrium toward, on average, more open conformations.

##### Exploring the Functional Significance of the Landscape by Pressure Perturbation

The PELDOR data imply an undulating conformational landscape for nNOS that can be perturbed by co-enzyme and CaM binding. We used high pressure to map the fitness (*i.e.* rate of electron transfer/enzyme turnover) onto the PELDOR-derived landscape. Hydrostatic pressure perturbs the equilibrium distribution of conformational states that comprise the free energy landscape for an enzyme-catalyzed reaction (23, 25, 26). As the effect of pressure on solvent and protein motions is governed by Le Chatelier's principle, an increase in pressure will favor more compact conformations that occupy smaller volumes, with concomitant effects on the observed rate of reaction. Unlike temperature, pressure only perturbs the equilibrium of states, and not the thermodynamics of the reaction. Studying enzyme kinetics under high pressure provides both structural and kinetic information; it allows assessment of whether there are multiple conformations, as well as the reaction rates catalyzed by the populated conformations (43). The effects of pressure on the quaternary structure of nNOS and nNOS reductase were studied prior to assessing outcomes on electron transfer (by variable pressure stopped-flow spectroscopy) and steady-state enzyme turnover.

##### Calmodulin Binding to nNOS under Pressure

**FIGURE 5.**
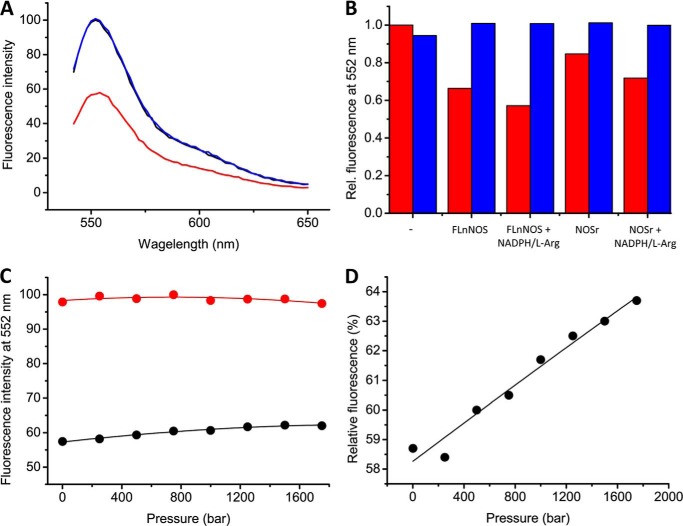
**Calmodulin binding to nNOS as a function of hydrostatic pressure.** All experiments were carried out at room temperature in buffer (pH 7.6) supplemented with 0.3 mm CaCl_2_, 2 mm l-arginine, and 100 μm NADPH, Ca^2+^ was removed by the addition of 0.5 mm EDTA, as described under “Materials and Methods.” *A*, fluorescence emission spectra (λ_exc_ = 536 nm) of CaM-T34C-A532 (*black line*), CaM-T34C-A532 plus full-length nNOS in the presence of Ca^2+^ (*red line*), and CaM-T34C-A532 plus full-length nNOS after the addition of EDTA (*blue line*). *B*, fluorescence quenching of CaM-T34C-A532 at 552 nm by full-length nNOS and nNOS_red_ in the resting state, or under turnover conditions (+NADPH/l-Arg), in the presence (*red*) or absence of Ca^2+^ (*blue*). *C*, pressure-dependent changes in fluorescence intensity of CaM-T34C-A532 upon binding of nNOS under turnover conditions, in the presence of Ca^2+^ (*black*) or EDTA (*red*). All data points were corrected for pressure-dependent changes in fluorescence in the absence of nNOS and fitted to Equation 2. *D*, relative fluorescence intensity of CaM-T34C-A532 in the presence of full-length nNOS and Ca^2+^ *versus* the same sample in the absence of Ca^2+^, as a function of hydrostatic pressure.

Fluorescence quenching was used to study the binding of CaM to nNOS under high pressure conditions. A variant form of CaM (CaM-T34C) was labeled with the fluorescent dye Atto532-maleimide to produce the mono-labeled conjugate CaM-T34C-A532. CaM-T34C-A532 was incubated with nNOS under turnover conditions (*i.e.* with excess l-arginine and NADPH) while increasing the hydrostatic pressure. Under binding conditions (*i.e.* in the presence of excess Ca^2+^) at atmospheric pressure, a maximum of 42% fluorescence quenching of CaM-T34C-A532 was observed, which is attributed to energy transfer of Atto532 emission to nNOS (Fig. 5*A*). On removing Ca^2+^ (by addition of excess EDTA) the maximum level of fluorescence is restored to that of an equivalent concentration of CaM-T34C-A532 alone, indicating release of CaM-T34C-A532 from nNOS. The level of quenching induced by binding of full-length nNOS to CaM-T34C-A532 was higher than that of nNOS_red_, because the heme absorbance at about 550 nm overlaps with the donor fluorophore emission (Fig. 5*B*). This is in addition to absorbance overlap with the blue (protonated) flavin semiquinones in both nNOS reductase and full-length nNOS during turnover. Fig. 5, *C* and *D*, show the pressure dependence of the fluorescence intensity of CaM-T34C-A532 in the presence of full-length nNOS. As pressure is increased, the fluorescence quenching under binding conditions (*i.e.* in the presence of excess Ca^2+^) decreased slightly from 42% at 1 bar to 36% at 1750 bar (after correction for pressure dependent changes of the “unbound state”). This relatively small difference indicates that CaM remains mostly bound to nNOS at high pressure, suggesting that nNOS is structurally intact at the quaternary level across the investigated pressure range. CaM also binds to monomeric nNOS_red_, so this assay does not provide direct information about nNOS dimerization as a function of pressure.

##### Flavin Reduction in nNOS as a Function of Pressure

**FIGURE 6.**
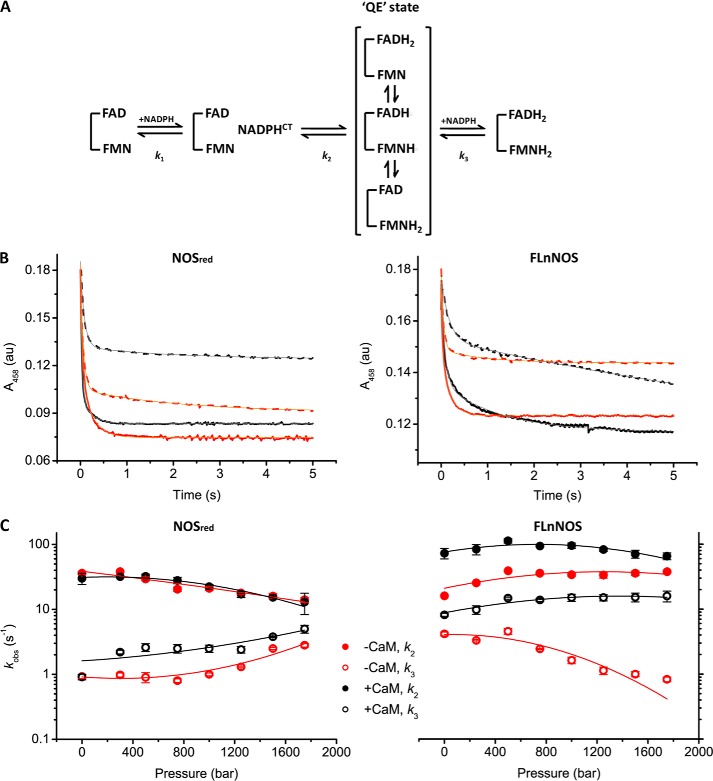
**The hydrostatic pressure dependence of flavin reduction in nNOS.** *A*, simplified scheme of the reductive half-reaction of nNOS. Reduction of nNOS by NADPH consists of at least three phases relevant for catalysis, with each intermediate comprising a number of different enzyme species. The first phase predominantly represents the rapid formation of the NADPH-enzyme charge transfer (*CT*) complex (*k*_1_ >500 s^−1^). The second phase (*k*_2_) predominantly represents the formation of the two-electron reduced species, or quasi-equilibrium (*QE*) state, and the third phase (*k*_3_) predominantly represents further reduction to the four-electron reduced species (27, 44). *B*, typical transients obtained at 458 nm upon reduction of nNOS_red_ (*left panel*) and full-length nNOS (*right panel*) in the presence (*black*) or absence (*red*) of CaM at low (1 bar, *solid lines*) and high pressure (1500 bar, *dashed lines*). Because the first phase is not observed in this study due to dead-time limitations of the high pressure stopped-flow instrument, transients were fitted to a two-exponential equation (*thin gray* and *orange lines* for CaM^+^ and CaM^−^, respectively). *C*, the hydrostatic pressure dependence of flavin reduction kinetics in nNOS_red_ (*left panel*), and full-length nNOS (*right panel*) measured at 25 °C in the presence or absence of CaM. The *error bars* represent the S.D. calculated from at least three individual transients. *Solid lines* represent fits to Equation 2 and the Δ*V*^‡^ and Δβ^‡^ values are given in Table 2.

Reduction of nNOS reductase by NADPH is complex and consists of at least three distinct kinetic phases when flavin reduction is monitored by stopped-flow spectroscopy (27, 28). Each spectral “intermediate” comprises a mixture of different enzyme species dictated by the thermodynamic equilibrium (27, 28) (Fig. 6*A*). The first phase (*k*_1_ >500 s^−1^) predominantly represents rapid formation of a NADPH-enzyme charge-transfer complex, but in the studies reported here this first phase is not observed due to dead-time limitations (5 ms) of the high pressure stopped-flow instrument. The second phase (*k*_2_) represents hydride transfer from NADPH to FAD yielding FADH_2_, and subsequent internal electron transfer to establish a “quasi-equilibrium” (QE) of two-electron reduced species (FADH_2_-FMN; FADH^•^-FMNH^•^; FAD-FMNH_2_), whose distribution is dictated by the relevant cofactor reduction potentials. This kinetic scheme is analogous to that developed for CPR, a close relative of nNOS (44). The third phase (*k*_3_) represents further reduction of the enzyme to the four-electron level (FADH_2_-FMNH_2_) following a second hydride transfer reaction from NADPH. Overall, NADP^+^ release is the limiting step for the flavin reduction chemistry in nNOS/nNOS reductase (27, 45).

The pressure dependence of flavin reduction in nNOS was measured anaerobically by mixing oxidized nNOS with excess NADPH in the high pressure stopped-flow instrument (Table 2). Flavin reduction was monitored by following the decrease in absorbance at 458 nm (a heme isosbestic point) at pressures ranging from 1 to 1750 bar. The observable kinetic phases (*k*_2_ reporting predominantly on 2 electron reduction to the QE state, and *k*_3_ reporting on 4 electron reduction) were analyzed by fitting to a two-exponential function. Typical transients obtained at low and high pressure for nNOS_red_ and full-length nNOS in the presence and absence of CaM are shown in Fig. 6*B*.

**TABLE 2:** **Pressure dependence of the observed rates** High-pressure fit parameters for the analysis of the observed rate constants for steady-state NADPH oxidation (ss), NO formation (NO), superoxide formation (O_2_^⨪^), and high-pressure stopped-flow fit parameters for pre-steady-state flavin reduction (*k*_2_) and (*k*_3_).

	*k*_0_	Δ*V*^‡^	Δβ^‡^
	*s*^−*1*^	*cm^3^ mol*^−*1*^	*cm^3^ mol*^−*1*^ *kbar*^−*1*^
**FLnNOS**			
SS	0.08 ± 0.01	−32.7 ± 3.6	−19.9 ± 3.4
O_2_^⨪^	0.11 ± 0.01	−52.9 ± 4.3	−36.5 ± 3.8
*k*_2_	20.7 ± 4.0	−23.5 ± 10.9	−18.6 ± 10.9
*k*_3_	4.2 ± 0.6	−0.6 ± 14.9	−38.1 ± 24.9

**FLnNOS + CaM**			
SS	1.3 ± 0.1	8.6 ± 4.2	−4.9 ± 5.4
NO	2.4 ± 0.1	38.7 ± 7.5	26.8 ± 11.2
O_2_^⨪^	0.22 ± 0.01	−3.7 ± 2.4	−2.7 ± 2.6
*k*_2_	75.3 ± 7.6	−18.3 ± 6.5	−24.7 ± 7.2
*k*_3_	8.7 ± 1.0	−21.5 ± 6.5	−15.6 ± 6.5

**nNOS_red_**			
*k*_2_	38.6 ± 3.2	13.6 ± 7.1	−2.0 ± 9.3
*k*_3_	0.9 ± 0.2	8.7 ± 12.6	29.0 ± 12.1

**nNOS_red_ + CaM**			
*k*_2_	31.1 ± 1.6	−4.1 ± 4.1	−21.6 ± 5.3
*k*_3_	1.6 ± 0.5	7.0 ± 14.9	9.7 ± 13.9

The hydrostatic pressure dependence of flavin reduction in nNOS_red_ is shown in Fig. 6*C* (*left panel*). Regardless of the presence of CaM, *k*_2_ decreases with pressure, whereas *k*_3_ increases with pressure. These pressure-related changes to *k*_2_ and *k*_3_ indicate a change in the conformational distribution across the energy landscape. For *k*_2_, the trends may represent a pressure-dependent separation of the FAD and FMN cofactors, slowing down internal electron transfer and further reduction by NADPH. Similar observations have been made with CPR (23). The pressure dependence of *k*_2_ was not affected by the presence or absence of CaM. The *k*_3_ trends are more complicated: this kinetic phase represents reduction of the flavin cofactors by a second hydride from NADPH (Fig. 6*A*), but also involves NADP^+^ release (the product of the first hydride transfer event), which is thought to be limiting for flavin reduction. Consequently, interpretation of the pressure dependence for *k*_3_ is less straightforward than for *k*_2_. A relatively small effect of CaM was observed on *k*_3_, perhaps implying that it influences to some extent the kinetics of NADP^+^ release. The small effect of CaM binding on *k*_3_ is consistent with previously reported studies with nNOS_red_ at 1 bar pressure (27).

In contrast to nNOS_red_, CaM has a major effect on the rate and pressure dependence of both *k*_2_ and *k*_3_ in full-length nNOS (Fig. 6*C*, *right panel*). The presence of the oxygenase domain alters the profile of the pressure dependence of flavin reduction compared with the nNOS_red_ situation, indicating there are functional consequences of remodeling the landscape through interactions of the oxygenase domain with other domains in nNOS. This remodeling of the overall landscape by the oxygenase domain is also evident from the PELDOR studies of full-length nNOS compared with nNOS_red_ (Fig. 4, *A* and *C*, compared with *F* and *H*, respectively). Although the pressure data does not provide direct information on the nature of the pressure-induced structural change, the pressure dependence of *k*_2_ is likely attributed to nNOS adopting altered populations, some of which have a higher probability of electron transfer as a result of optimizing distances between redox centers at high pressure. That *k*_3_ has markedly different pressure response profiles in the presence/absence of CaM suggests that NADP^+^ release, which is limiting for *k*_3_ (see above), might be enhanced in the presence of CaM. A more thorough analysis of the decay of the NADP^+^-enzyme charge-transfer complex is hampered by the small signal changes of the charge-transfer signature at 600 nm (27), a region where also the flavin semiquinones and heme show absorbance features.

##### Pressure Perturbation of nNOS Turnover

**FIGURE 7.**
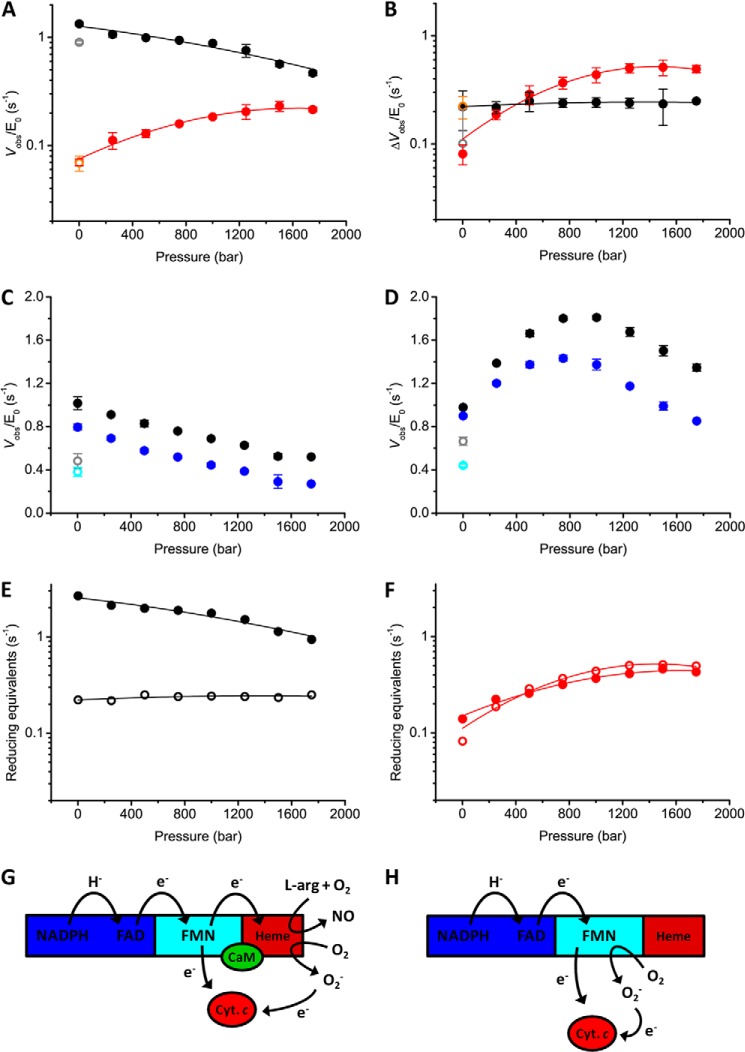
**The hydrostatic pressure dependence of NADPH turnover by full-length nNOS.** All reactions were performed under aerobic conditions in the presence of excess l-arginine and NADPH at pH 7.6 and at 25 °C as described under “Materials and Methods.” *A*, observed rate (*V*_obs_/*E*_0_) of NADPH turnover in the absence of external electron acceptors for nNOS plus (*black circles*) or minus CaM (*red circles*) *versus* hydrostatic pressure. The *error bars* represent the S.D. calculated from at least three measurements. The data are fitted to Equation 2 and the Δ*V*^‡^ and Δβ^‡^ values are given in Table 2. The *open gray* and *orange circles* represent the observed rates after de-pressurizing the sample for CaM^+^ and CaM^−^, respectively. *B*, observed difference in cytochrome *c* reduction rate (Δ*V*_obs_/*E*_0_) for nNOS plus (*black circles*) or minus CaM (*red circles*) measured in the absence and presence of superoxide dismutase. This figure was generated using the data of *panels C* and *D*. The pressure dependence of cytochrome *c* reduction and its inhibition by superoxide dismutase for full-length nNOS in the presence (*C*) or absence (*D*) of CaM. *Black circles*, rates observed in the absence of superoxide dismutase; *blue circles*, rates observed in the presence of superoxide dismutase. The *open gray* and *cyan circles* represent the observed rates after de-pressurizing the sample for superoxide dismutase^−^ and superoxide dismutase^+^, respectively. The *error bars* represent the S.D. calculated from 2 to 3 individual transients. Comparison of reducing equivalents originating from NADPH (*solid circles*) resulting in O_2_^⨪^ (*open circles*) in nNOS CaM^+^ (*E*) and CaM^−^ (*F*) shows that in the absence of CaM all electrons from NADPH result in the formation of reactive oxygen species. The schematic representations show the various productive and non-productive electron transfer reactions in nNOS CaM^+^ (*G*) and CaM^−^ (*H*).

**FIGURE 8.**
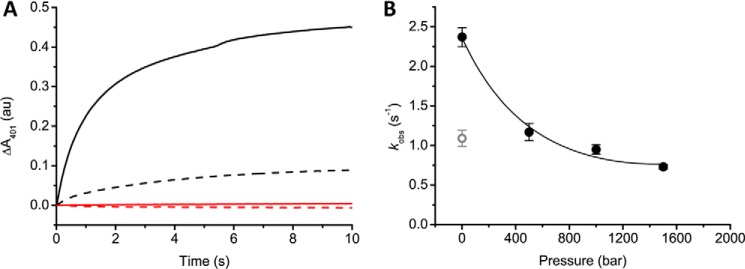
**The hydrostatic pressure dependence of single-turnover NO formation by full-length nNOS.** NO formation was detected using oxyhemoglobin as described under “Materials and Methods.” *A*, progress curves, corrected for background absorbance, obtained at 401 nm for nNOS in the presence of CaM at 1 bar (*black*, *solid line*), 1500 bar (*black*, *dashed line*), and nNOS in the absence of CaM at 1 bar (*red*, *solid line*) and 1500 bar (*red*, *dashed line*). *B*, pressure dependence of NO formation for full-length nNOS in the presence of CaM. The *open gray circle* represents the observed rate after de-pressurizing the sample. The *error bars* represent the S.D. calculated from three individual transients.

Increasing hydrostatic pressure will lead generally to more compact states of NOS achieved by remodeling the distribution of conformational states across the landscape. Where this compaction leads to a shortening of electron transfer distances one would expect faster electron transfer rates (assuming no change in driving force or reorganizational energy); predicting effects on ligand binding/release is more complicated as this will depend on local structural changes in the ligand binding site induced by pressure. The steady-state activity of nNOS as a function of hydrostatic pressure was investigated to provide additional insight into the functional consequences of remodeling the conformational landscape. Steady-state turnover measurements were carried out under aerobic conditions with excess NADPH and l-arginine at 25 °C in a high pressure stopped-flow instrument. At atmospheric pressure (1 bar) *k*_cat_ values (determined by following the rate of depletion of NADPH at 340 nm) for full-length nNOS are 1.1 and 0.15 s^−1^ in the presence or absence of CaM/Ca^2+^, respectively. This ∼10-fold stimulation of NADPH turnover through CaM binding corresponds to previously published values (46, 47). With increasing pressure, *k*_cat_ values for nNOS in the presence of CaM were found to progressively decrease; the opposite effect was observed for nNOS in the absence of CaM (Fig. 7*A*). At elevated pressure (1750 bar) *k*_cat_ values for nNOS in the presence/absence of CaM begin to converge (0.5 and 0.3 s^−1^ plus/minus CaM, respectively; Fig. 7*A*). This suggests that increasing pressure reduces the dependence on CaM under turnover conditions. As such, at a phenomenological level, pressure appears to mimic the effects of CaM binding by re-modeling the nNOS landscape, with the functional consequence of optimizing the rate of NADPH turnover. The overall rate-limiting step in NO synthesis is electron transfer from the FMN domain to the heme oxygenase domain (13, 29). It is proposed that this step is activated by CaM and coupled to large-scale motions of the FMN domain (2, 10). To test whether pressure can also induce NO formation in nNOS in the absence of CaM/Ca^2+^, we measured single turnover NO formation as a function of pressure, using oxyhemoglobin as a NO scavenger (Fig. 8). Where the NO formation by nNOS in the presence of CaM shows a comparable pressure dependence as NADPH oxidation, *i.e.* a decrease with increasing pressure (Fig. 8*B*), NO formation could not be detected for nNOS in the absence of CaM at atmospheric pressure (1 bar), or elevated pressure (1500 bar) (Fig. 8*A*). This observation poses the question: where do the electrons go in nNOS in the absence of CaM when NADPH depletion is stimulated by high pressure?

NO is normally produced by nNOS through a tightly coupled electron transfer chain, *i.e.* vectorial electron transfer from NADPH → FAD → FMN → heme in the absence of leakage to molecular oxygen. In principle, uncoupling of this chain can lead to the production of reactive oxygen species (*e.g.* superoxide, O_2_^⨪^). The heme oxygenase domain and the flavin reductase domain of nNOS are capable of generating O_2_^⨪^ (48, 49). Also, the reaction of nNOS with cytochrome *c* is, in part, attributed to O_2_^⨪^ formation, which rapidly reduces the heme in cytochrome *c* (50). A possibility we considered is that the pressure-dependent increase in the rate of NADPH turnover for nNOS (without CaM) is attributed to increased O_2_^⨪^ formation. We used the cytochrome *c* reduction assay to detect superoxide generated by nNOS. The reaction was performed in the absence and presence of superoxide dismutase to verify that the electron for the reduction of cytochrome *c* originated from O_2_^⨪^. Superoxide dismutase reacts rapidly with O_2_^⨪^, converting it into hydrogen peroxide and oxygen. Increased rates of O_2_^⨪^ formation will therefore decrease the cytochrome *c* reduction rate when superoxide dismutase is present. Both nNOS plus and minus CaM were shown to generate superoxide using this assay (Fig. 7, *C* and *D*, respectively). The pressure dependence of O_2_^⨪^ formation catalyzed by nNOS ± CaM is shown in Fig. 7*B*. The rate of O_2_^⨪^ formation increases with pressure for nNOS in the absence of CaM (*V*_obs_/[*E*]_0_ increases from 0.08 s^−1^ at 1 bar to 0.5 s^−1^ at >1250 bar), which is similar to the pressure dependence of NADPH depletion (Fig. 7*A*). When we compare the NADPH turnover rate with O_2_^⨪^ formation, we need to take the reaction stoichiometry into account; for each hydride transfer from NADPH (two electron reaction), two O_2_^⨪^ molecules (one electron reaction) are formed. On overlaying the number of electrons donated by NADPH with the number of electrons accepted by oxygen, it becomes clear that for nNOS in the absence of CaM ∼100% of electrons originating from NADPH are transferred to O_2_^⨪^ over the pressure range studied (Fig. 7*F*). In contrast, pressure did not affect the rate of O_2_^⨪^ formation for nNOS in the presence of CaM. The rate of O_2_^⨪^ formation over the pressure range is constant at ∼0.2 s^−1^ (Fig. 7*E*). However, pressure does induce some uncoupling of the reaction, even in the presence of CaM; at 1750 bar ∼25% of the electrons originating from NADPH result in O_2_^⨪^ formation, compared with <10% at atmospheric pressure.

In the presence of CaM, electrons derived from NADPH are transferred to the heme oxygenase domain, resulting in the formation of NO, but in the absence of CaM all electrons result in O_2_^⨪^ production. We have shown that electron transfer to the heme does not occur in the absence of CaM (NO formation is not detected at low or high pressure; Fig. 8*A*), indicating that all O_2_^⨪^ in the absence of CaM is generated through flavin chemistry (Fig. 7*G*). We infer therefore that CaM protects nNOS from generating these reactive oxygen species, although to some extent this becomes less significant at high pressure. The different pressure dependence of the O_2_^⨪^ production in the presence/absence of CaM (Fig. 7, *E* and *F*, respectively), and the fact that electron transfer from FMN to the heme oxygenase domain does occur in the presence of CaM, probably reflects the fact that O_2_^⨪^ formation in the presence of CaM is predominantly through the oxygenase domain chemistry, and not flavin chemistry as is the case with NOS in the absence of CaM (Fig. 7*H*).

We note that the pressure-dependent decrease in overall turnover (NADPH oxidation and NO formation) for nNOS CaM^+^ is accompanied by a pressure-dependent increase in the rates of flavin reduction and inter-flavin electron transfer (Fig. 6*C*, *right panel*). This demonstrates an interesting opposing effect of pressure: optimizing flavin reduction and inter-flavin electron transfer presumably requires enrichment of those conformations that enable closer proximity of the flavin cofactors. However, by bringing the FMN domain closer to the FAD domain, this presumably translates to a less favorable FMN-oxygenase domain interaction. The FMN-oxygenase domain geometry controls the overall rate-limiting step in catalysis, accounting for the loss of catalytic activity at higher pressure.

##### The Key Role of CaM in Conformational Sampling, Vectorial Electron Transfer, and Suppression of “Off Pathway” Redox Chemistry

**FIGURE 9.**
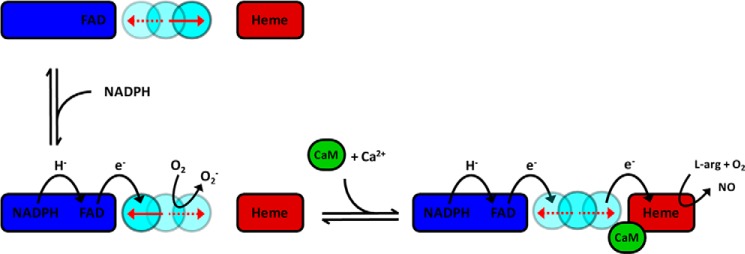
**Schematic representation of the conformational equilibria in nNOS during catalysis.** The FAD-binding subdomain is shown in *blue*, and the mobile FMN-binding module is shaded in *cyan*. The reductase domain of nNOS in the resting state adopts mostly open conformations with relatively large inter-flavin distances, which shift toward the population of more closed conformations with shorter inter-flavin distances upon binding of NADPH. In the absence of CaM nNOS predominantly populates conformations with shorter inter-flavin distances, *i.e.* conformations favorable for FAD to FMN electron transfer with some “leakage” of electrons to molecular oxygen or other external electron acceptors. In the presence of CaM the conformational equilibrium shifts toward on average intermediate inter-flavin distances, consistent with the sampling of conformations compatible with both inter-flavin electron transfer and FMN to heme electron transfer. During catalysis the FMN subdomain interacts alternately with the FAD subdomain and heme oxygenase domain, resulting in NO formation. The *red arrows* indicate the predominant “position” of the FMN subdomain. Note that electrons are transferred from the FMN subdomain of one monomer to the heme oxygenase domain of the other monomer, for clarity only one monomer is shown.

Current models, based on static crystal structures of the isolated NOS domains, in which CaM binding activates NOS through unlocking of the FMN domain (open and closed conformations), are clearly too simplistic for describing NOS function (2, 8, 10, 51). Our PELDOR and variable pressure kinetic studies indicate a more complex distribution of conformational states that can be functionally remodeled by ligand (NADPH, CaM) binding (Fig. 9). The PELDOR data indicate that in nNOS the reductase domain adopts predominantly more open conformations with relatively large inter-flavin distances, which shift toward a population of more closed conformations on binding NADP(H). In the absence of CaM, nNOS populates more closed conformations; this inhibits FMN to heme electron transfer and enables electrons derived from NADPH oxidation to flow to the flavin cofactors and ultimately molecular oxygen (the uncoupled superoxide generation pathway). In the presence of CaM, the conformational equilibrium is shifted to more open reductase domain conformations. This is consistent with access of the FMN domain to both the FAD and oxygenase domains through conformational sampling, enabling more efficient electron transfer from NADPH to the oxygenase domain. The pressure perturbation studies demonstrated that CaM is a crucial effector of electron transfer by perturbing the landscape in a way that enables efficient electron transfer between both flavins and also across the FMN-oxygenase domain interface. In facilitating electron transfer from NADPH to the oxygenase domain, CaM also prevents leakage of electrons from the NADPH to heme electron transfer pathway by suppressing flavin-dependent oxidase chemistry. CaM is therefore a key component in driving vectorial electron transfer by minimizing “off pathway” transfer of electrons to oxygen through flavin oxidase chemistry. The inferred requirement for extensive conformational sampling to enable electron transfer in NOS from the reductase domain to the oxygenase domain explains why electron transfer from FMN to heme is relatively slow, and thus limits overall turnover. Our studies have therefore provided detailed insight into the nature of the free energy landscape of NOS, and its remodeling by CaM and NADP(H) binding, which will likely impact on the electron transfer and catalytic properties of the enzyme.

## Footnotes

3 The abbreviations used are:

CaM: calmodulin
nNOS: neuronal NOS
eNOS: endothelial NOS
iNOS: inducible NOS
CPR: cytochrome P450 reductase
PELDOR: pulsed electron-electron double resonance
nNOS_red_: neuronal NOS reductase domain.
